# GENOME-WIDE LINKAGE ANALYSIS IDENTIFIES A NOVEL LOCUS FOR LONGITUDINAL GAIT SPEED CHANGE WITH AGING

**DOI:** 10.1093/geroni/igz038.2270

**Published:** 2019-11-08

**Authors:** Adam J Santanasto, Mary K Wojczynski, Ryan K Cvejkus, Bharat Thyagarajan, Kaare Christensen, Nicole Schupf, Ping An, Joseph M Zmuda

**Affiliations:** 1 University of Pittsburgh, Pittsburgh, Pennsylvania, United States; 2 Department of Genetics Washington University in St. Louis, Saint Louis, Missouri, United States; 3 Department of Epidemiology, University of Pittsburgh; Pittsburgh, Pennsylvania, United States; 4 Department of Laboratory Medicine and Pathology, University of Minnesota; Minneapolis, Minnesota, United States; 5 Danish Aging Research Center, University of Southern Denmark, Odense C, Denmark; 6 Columbia University, Columbia University, United States; 7 St. Louis, Washington University, Missouri, United States

## Abstract

Gait speed is an indicator of health and function with aging. The potential genetic contributions to gait speed and its decline with aging are not well characterized. We sought to better quantify the genetic contributions to and identify potential genes and genetic variants underlying change in gait speed among older adults. To accomplish these aims, we used data from 2379 individuals belonging to 509 families in the Long Life Family Study (mean age 64 ± 12, range 30–110 years; 45% men). Gait-speed was measured over 4 meters at baseline and after an average of 7±1.1 years. Quantitative trait linkage analyses were completed using pedigree-based maximum-likelihood methods with logarithm of the odds (LOD) scores > 3.3 indicating genome-wide significance. We also performed linkage analysis in the top 10% of families contributing to LOD scores to allow for heterogeneity among families (HLOD). Data were adjusted for age, sex, height and field center. At baseline, 26.9% of individuals had “low” gait-speed <1.0 m/s (mean: 1.1±0.2 m/s) and gait speed declined at a rate of -0.02±0.03 m/s per year (p<0.0001). Baseline and change in gait-speed were significantly heritable (h2 = 0.24-0.32, p<0.05). We did not find significant evidence for linkage for baseline gait speed; however, we identified a potentially novel locus for change in gait speed on chromosome 16p (LOD 4.2). A subset of 21 families contributed to this linkage peak (HLOD = 6.83). Sequence analysis of the chromosome 16 region may yield new insight on the biology of age-related mobility decline.

